# How is emotional resonance achieved in storytellings of sadness/distress?

**DOI:** 10.3389/fpsyg.2022.952119

**Published:** 2022-09-29

**Authors:** Christoph Rühlemann

**Affiliations:** Deutsches Seminar-Germanistische Linguistik, University of Freiburg, Freiburg, Germany

**Keywords:** storytelling interaction, multimodality, emotion contagion, sadness/distress, gesture expressivity, electrodermal activity

## Abstract

Storytelling pivots around stance seen as a window unto emotion: storytellers project a stance expressing their emotion toward the events and recipients preferably mirror that stance by affiliating with the storyteller’s stance. Whether the recipient’s affiliative stance is at the same time expressive of his/her emotional resonance with the storyteller and of emotional contagion is a question that has recently attracted intriguing research in Physiological Interaction Research. Connecting to this line of inquiry, this paper concerns itself with storytellings of sadness/distress. Its aim is to identify factors that facilitate emotion contagion in storytellings of sadness/distress and factors that impede it. Given the complexity and novelty of this question, this study is designed as a pilot study to scour the terrain and sketch out an interim roadmap before a larger study is undertaken. The data base is small, comprising two storytellings of sadness/distress. The methodology used to address the above research question is expansive: it includes CA methods to transcribe and analyze interactionally relevant aspects of the storytelling interaction; it draws on psychophysiological measures to establish whether and to what degree emotional resonance between co-participants is achieved. In discussing possible reasons why resonance is (not or not fully) achieved, the paper embarks on an extended analysis of the storytellers’ multimodal storytelling performance (reenactments, prosody, gaze, gesture) and considers factors lying beyond the storyteller’s control, including relevance, participation framework, personality, and susceptibility to emotion contagion.

## Introduction

Storytelling is powered by emotion—this has been clear from the early days of Discourse Analysis when Labov declared evaluation storytelling’s “raison d’être” (Labov, 1972: 366). He explained that the role of evaluation is to “say to us: this was terrifying, dangerous, weird, wild, crazy; or amusing, hilarious, wonderful; more generally, that it was strange, unusual, or uncommon” (Labov, 1972: 371). While Labov’s discovery of evaluation as key to storytelling was a milestone in the quest for a fuller understanding of why we do storytelling, evaluation was merely seen as a function of the speaker’s *linguistic* story design (as evidenced by the use of direct speech, comparators, evaluative terms, etc.). Labov did not have any concern for non-linguistic evaluative resources deployed by the speaker. Indeed, Labovian stories are maximally decontextualized (Edwards, 1997). Beside the interviewer’s elicitation question (e.g., “Were you ever in a situation where you were in serious danger of being killed?”), the story transcripts report only the storyteller’s words. Furthermore, Labov was not concerned at all with evaluation as a function of the speaker-recipient interaction (Schegloff, 1997: 101). As a result, Labovian analyses fail to grasp the *co-constructive* nature of storytelling as a “tool for collaboratively reflecting upon specific situations and their place in the general scheme of life” (Ochs and Capps, 2001: 2). Labovian analyses also fail to elucidate the ways in which story recipients become co-authors by “provid[ing], elicit[ing], criticiz[ing], refut[ing], and draw[ing] inferences” (Ochs and Capps, 2001: 2–3) from the storytelling—inferences which benefit not only cognition but also the “emotional dimension to linguistic communication—the less epistemic, or ‘lower’ cognitive side” (Wharton et al., 2021: 269). This disregard for non-linguistic resources, the speaker-recipient interaction, and the co-construction of narrative changes radically in Conversation Analysis (CA), a field that entertains a decidedly multimodal and interactional view of storytelling. This view rests on three pillars. Central to it is the notion of ‘stance, CA’s rough equivalent of Labov’s ‘evaluation.’ Stance is defined as “the teller’s affective treatment of the events (…) communicated explicitly or implicitly” (Stivers, 2008: 37).

First, stance-taking is seen as a reciprocal process involving both storyteller and story recipient: the storyteller “takes a stance toward what is being reported and makes the taking of a stance by the recipient relevant” (Stivers, 2008: 32). On this view, stance is not only the property of the storyteller’s discourse but emergent in the storyteller-recipient interaction. The interactionally constructed stance is preference-operated: preferably, the recipient’s stance *mirrors* the storyteller’s stance, thus creating affiliation (Stivers, 2008). In other words, underlying the CA model of storytelling is (the expectation of) stance alignment between storyteller and recipient, or what could be called stance contagion. It typically occurs at the climax of the story where the most intensive display of affiliation is expected (cf. Selting, 2010; Kupetz, 2014).

Second, affective stance is conveyed not only through Labov’s verbal means (s. above) but also *performed multimodally* “through intonation, gesture, and body posture” (Goodwin et al., 2012: 16; cf. also Selting, 2010, 2012; Couper-Kuhlen, 2012) and gaze behavior (e.g., Rühlemann et al., 2019).

Third, the display of stance is at the same time a display of emotion: “there is no doubt that the scope of an emotion is *not* restricted to the individual who displays it. By virtue of their systematic expression on the face (and elsewhere, such as in prosody) emotions constitute public forms of action” (Goodwin et al., 2012: 17, emphasis in original). As a result, in CA, “the study of affectivity and emotion in social interaction has developed from a sideshow to a main field of interest in recent years” (Kupetz, 2014: 4). Given that emotion is the central concept of this research, an excursus is warranted.

Defining emotion is a thorny issue that neither Goodwin et al. nor Kupetz address. Systematically reviewing it and distinguishing emotions from related concepts such as affect, mood, or feeling is far beyond the aims of this paper. Broadly, a distinction can be made between constructivist and essentialist accounts of emotion, the former being based on prediction, the latter on reaction.

The constructivist view on emotion proposes that emotions are constructed by the individual’s brain drawing on past experience and predictive modeling in the service of allostasis. Allostasis is the balancing act of how the brain regulates the body by *anticipating* its needs and satisfying them preemptively (Sterling, 2012). To anticipate needs, the brain runs internal models (‘embodied simulations’) that include the statistical regularities of the body’s internal milieu and those of the sensory environment. The key question guiding the simulations is ‘what past experience is the new sensory input most similar to?’ (Barrett, 2016: 7).^1^ As experiences are implemented in concepts, the brain “continually constructs concepts and creates categories to identify what the sensory inputs are […]. When the internal model creates an emotion concept, the eventual categorization results in an instance of emotion” (Barrett, 2016: 13). Given that each individual brain draws on different past experiences to guide its predictions, the constructivist account stresses and helps explain the massive variability of emotion perception and expression across individuals.

The essentialist account of emotion assumes that, not (internal) predictions, but (internal or external) stimuli trigger emotions. A proponent of the essentialist view of emotion is, for example, Scherer (2005). He proposes a ‘component process’ view of emotion, defining emotion “as an episode of interrelated, synchronized changes in the states of all or most of the five organismic subsystems in response to the evaluation of an external or internal stimulus event as relevant to major concerns of the organism” (Scherer, 2005: 697). The components of an emotion include cognition (how the emotion is appraised), neurophysiology (how the emotion is indexed in bodily symptoms), motivation (what action tendencies it gives rise to), motor expression (how the emotion is expressed facially or vocally), and subjective feeling (what experience the emotion prompts). This present research is centrally concerned with the neurophysiological and the motor expression components: the participants’ Electrodermal Activity (EDA) (s. below) provides a window onto neurophysiological changes induced by the emotions conveyed or mirrored, while the participants’ verbal, vocal, and kinesic behavior indexes how their emotions are expressed. These emotion components are not affected by the controversy between constructivism and essentialism. For even if emotions are the result not of a stimulus but of a prediction of that stimulus, there will still be changes in neurophysiology and motor expressions as the brain adjusts to it.

Further, emotions are at least partly universal. Ekman and O’Sullivan (1991: 176) discuss evidence for the cross-cultural recognition of at least six emotions expressed *via* facial actions: happiness, anger, fear, sadness/distress, surprise, and disgust (cf. also Ekman, 2016). Far more emotions have been identified beyond that universal core; Ekman (1999) specifies 15 basic emotions, which may have evolved “for their adaptive value in dealing with fundamental life tasks” (Ekman, 1999: 46) such as loss, danger, achievement, or fulfillment. Related emotions exhibit variation. Ekman and Friesen (1978), for example, found 60 related but visually different facial expressions of anger sharing certain core configurational properties. These differences give rise to different emotional ‘hues’; in the case of anger, these include, for example, resentment, indignation, outrage, vengeance and others (Ekman, 1993: 386). Considering this variation, Ekman argues that emotions are best thought of as emotion families, where “each emotion family can be considered to constitute a theme and variations.”

But emotions also have key interpersonal and interactional implications. Darwin proposed the idea that facial expression evolved to elicit response in conspecifics (Darwin, 1872). Consistent with this view, a large body of research suggests that the display of emotion can stimulate reciprocal emotional response (cf. Keltner and Ekman, 2000 and references therein). For example, observers responded with fear-related reactions to facial expression of anger, even when presented subliminally, i.e., below the observer’s conscious awareness (Esteves et al., 1994). The emotion-emotion response relationship is also key to empathy. Empathy is a multidimensional phenomenon comprising both cognitive empathy, “the intellectual/imaginative apprehension of another’s mental state” (Lawrence et al., 2004: 911), a capacity that largely overlaps with Theory of Mind (cf. Enfield and Levinson, 2006), and affective empathy, that is, emotional responsiveness to another’s emotional state. Affective empathy can occur in two different incarnations, parallel and reactive. A parallel empathic response in the other matches that of the observed (your fear becomes my fear), a reactive empathic response involves a complimentary emotion in the other such as sympathy or compassion (e.g., Ekman, 2014) designed to alleviate the observed’s sadness, suffering, or distress. Crucially, not every reactive emotional response is considered truly empathic. For example, Eisenberg et al. (1989) have shown that facial expression of distress may elicit two different emotional reactions in the observer: sympathy and personal distress. Eisenberg et al. define sympathy as “an other-oriented emotional reaction” (Eisenberg et al., 1989: 55) that is guided by the “altruistic goal of alleviating the other’s distress or need, even if it is easy to escape contact with the needy other” (Eisenberg et al., 1989: 55–56). If the other’s distress evokes sympathy in the observer, the observer is said to emotionally resonate (Ekman, 2014) — emotional contagion is achieved. Personal distress, by contrast, is a “self-oriented, aversive emotional reaction such as anxiety or discomfort” (Eisenberg et al., 1989: 55).^2^ Personal distress in response to other’s sadness/distress can still lead to attempts by the observer to relieve the needy other’s distress but these attempts are egoistically motivated (Batson, 1987). The two emotional reactions toward sadness/distress are differentially correlated with a psychophysiological metric, heart rate: while the sympathetic observer’s heart rate decreases, the heart rate of the observer with a self-focused personal distress reaction accelerates (Eisenberg et al., 1989 and references therein; cf. also Keltner and Ekman, 2000; Ekman et al., 1983).

Emotions tend to be contagious (see Doherty, 1997 and references therein). Hatfield et al. observed a tendency for people in social interaction not only to continuously and non-consciously monitor and but also to mimic the other’s emotional expression and to “synchronize [their] expressions, vocalizations, postures, and movements […] and, consequently, to converge emotionally” (Hatfield et al., 1994: 5). However, contagion is not automatic. One person’s emotion display may not always trigger an emotional response. Observers can remain uninfected and relatively distanced when the way they empathize with the needy other is restricted to recognizing (but not resonating with) the other’s emotional state — a reaction called emotion recognition (Ekman, 2014) or cognitive empathy (Lawrence et al., 2004). Finally, to blur the boundaries between emotion and non-emotion even further, there is not a simple one-to-one relation between emotion experience and emotion appearance (on the face or elsewhere). Not only can emotional states be ‘false’ (Ekman, 1993: 390) that is, ‘staged’ either for deception or social purposes (e.g., the social smile), there can also be emotions whose expression is inhibited and that therefore remain socially undetected (Ekman, 1999: 48).

To finally return to CA’s three-fold view of storytelling and to stitch the three pieces together, storytellers seek to transmit their stance or emotion toward the story events to the story recipient, thus sharing not only information about events but, more importantly, emotions. The true *raison d’être* of storytelling, then, may be to bring about emotion contagion (cf. Hatfield et al., 1994; Doherty, 1997). That tellers *actively work* toward achieving emotion contagion transpires from storytellings where recipients behave non-affiliatively despite the storyteller’s use of affect-laden stance devices around the Climax. Storytellers have been shown to *pursue* affiliation by recycling the Climax with increased use of multimodal resources (e.g., Couper-Kuhlen, 2012; Peräkylä and Ruusuvuori, 2012; Peräkylä et al., 2015; Rühlemann, 2020).

Intricate ways in which affiliation in storytelling is correlated with emotion arousal have recently been demonstrated in Physiological Interaction Research (e.g., Peräkylä et al., 2015). This new field examines physiological processes underpinning affiliation based on psychophysiological measures associated with emotion (Scherer, 2005), especially EDA, a reliable indicator of emotional arousal, i.e., the intensifying activation of the autonomic nervous system associated with emotion (Peräkylä et al., 2015: 302). Research in this field has, for example, shown that displays of affiliation by recipients have the effect of decreasing the storyteller’s arousal and increasing arousal in the story recipient while non-displays of affiliation lead to increased arousal in the storyteller (Peräkylä et al., 2015: 302).

The present research connects to this approach. Specifically, this paper asks: How is emotional resonance achieved in storytellings of sadness/distress? It aims to identify factors that facilitate emotion contagion and factors that impede it. Given the complexity and novelty of this question, this study is designed as a *pilot study* to scour the terrain and stake out interim directions before a larger study is undertaken. While the data base is small, comprising two storytellings of sadness/distress, the methodology used is expansive: it includes CA methods to transcribe and analyze interactionally relevant aspects of the storytelling interaction; it draws on psychophysiological measures to establish whether and to what degree emotional resonance between co-participants is achieved. In identifying possible reasons why resonance is (not or not fully) achieved, the paper embarks on an extended analysis of the storytellers’ multimodal storytelling performance (reenactments, prosody, gaze, gesture) examining key resources on all three major multimodal levels (verbal, vocal, and kinesic) are examined.

On the verbal level the focus is on quotation, alternatively referred to as ‘(free) direct speech’ (e.g., Labov, 1972), ‘(re-)enactment’ (e.g., Holt, 2007), or ‘constructed dialog’ (e.g., Tannen, 1986). Quotations thrive in storytellings (Rühlemann, 2013; Stec et al., 2016). Contrary to traditional assumptions based on the verbatim hypothesis, Clark and Gerrig (1990) argue that quotations are selective depictions of utterances rather than faithful renditions, a point underscored by empirical research by Wade and Clark (1993). Arguably due to their character as depictions, quotation has been variously described as a site for theatricalization (Buchstaller, 2002) or dramatization (Mathis and Yule, 1994) creating involvement (Tannen, 1989) and adding liveliness (Groenewold et al., 2014). An essential component of ‘doing’ constructed dialog is a shift, in Goffman’s (1981) terms, from ‘author,’ someone “formulating his own text” (1981: 145), to ‘animator,’ “a body engaged in acoustic activity” (1981: 144). The reduced responsibility that comes with the switch into ‘animator’ has a relaxing effect allowing speakers to take greater liberties to perform normally dispreferred actions (cf. Goffman, 1981; Bergmann, 1993). Moreover, constructed dialog has been shown to invite heightened activation in the vocal and bodily channels by the speaker (e.g., Blackwell et al., 2015; Stec et al., 2016; Soulaimani, 2018). Also, constructed dialog is frequently mimicry (e.g., Mathis and Yule, 1994; Stenström et al., 2002: 112; Halliday and Matthiessen, 2004: 447). Mimicry in constructed dialog has an *alienating* effect: it creates ‘quotative distance’ (Stenström et al., 2002: 110) on the part of the mimicking speaker toward the mimicked person and reflects “disaffiliatory intentions” (Couper-Kuhlen, 1996: 367). Culpeper (2011) discusses mimicry as an impoliteness strategy on the grounds that it can be seen as “a caricatured re-presentation” (Culpeper, 2011: 161) or ‘echo’ of anterior discourse, “reflect[ing] the negative attitude of the echoer toward the echoed person” (Culpeper, 2011: 165). Constructed dialog is therefore a highly likely candidate resource for storytellers to display emotion and, as a result, to engender emotional responses in the recipients.

Among the vocal resources examined in this study are pitch and intensity. Pitch and intensity are elements of paralinguistic prosody, whose claviature enables speakers to “achieve an infinite variety of emotional, attitudinal, and stylistic effects” (Wennerstrom, 2001: 200). Paralinguistic prosody is “both pervasive and absolutely central to the organization of affective stance” (Goodwin et al., 2012: 22) as it creates the “presence of hearable emotion” (Goodwin et al., 2012: 22). For example, when speakers are being sarcastic, they tend to use “heavily intonated voice” (Eaton, 1988: 126). Wennerstrom (2001:201) observes that “speakers manipulate prosodic variables to enliven or intensify key elements of text.” Pitch and intensity are particularly emotion-related as primary means to achieve “attitudinal stance” (Biber et al., 1999: 967; a third emotion-laden parameter is elongation, as described in great detail in Goodwin et al. (2012); another is timbre (distribution of spectral energy) [cf. Liebenthal et al., 2016]). Initial evidence suggests that storyteller’s pitch and intensity increase over the course of storytellings and peak at Climax (Rühlemann, 2020; cf. also Selting, 2012).

The kinesic modality is no less indicative of emotion although “a thorough understanding of the role of body movement, posture and especially gesture in emotion communication is still lacking” (Dael et al., 2013: 642). Referring to manual gesticulation, Kendon notes that “speakers often use gestures for dramatic effect” (Kendon, 2004: 198) thereby implying an intention by storytellers to affect recipients emotionally. Gestures have been noted as displays of emotional states by numerous authors, including Goodwin et al. (2012: 16), Selting (2010, 2012), and Couper-Kuhlen (2012), to name but a few. Intriguing work by Dael et al. (2013) has raised the question as to what makes gestures ‘emotionally salient’ and argued for a dynamic rather than categorical/static view where the emotional expressiveness of a gesture depends not (only) on the kind of gesture but the dynamic properties (such as speed, force, size, etc.; see below) with which it is performed *in situ*.

As regards gaze, Kendon (1967) may have been the first to discover the connection between mutual eye gaze and emotion arousal; he argued that “the amount of mutual looking conversants will engage in can serve to regulate the level of shared emotional arousal within it” (Kendon, 1967: 42). In recent work on storytelling in triads, Rühlemann et al. (2019) observed that storyteller’s gaze alternation between recipients accelerates toward Climax and decelerates in Post-completion sequences; the authors concluded that “accelerated gaze alternation constitutes an indexical process drawing the recipients’ attention to the immediate relevance of stance display” (Rühlemann et al., 2019: 91).

## Data and methodology

### Data

The data for this study come from the Freiburg Multimodal Interaction Corpus (FreMIC) currently under construction at Freiburg University with funding from the DFG (project number 414153266^3^). At present, the corpus contains roughly 20 h of video-recordings of unscripted conversation in two- to three-participant settings.

The recordings have been transcribed in Elan (2019), a transcription tool for multimodal interaction research, using both orthographic and conversation-analytic transcription (e.g., Jefferson, 2004), the latter recording not only verbal content but also interactionally relevant details of sequencing (e.g., overlap, latching), temporal aspects (pauses, acceleration/deceleration), phonological aspects (e.g., intensity, pitch, stretching, and truncation), laughter and free-standing gestures. Moreover, the FreMIC data are consistently transcribed for quotations, both on a separate tier in ELAN to capture their durations as well as within the utterance tiers.

The task of annotating storytellings and their climaxes was shared by two independent raters and criteria-based. The criteria for story identification included the following: (i) shifts in body posture (Rossano, 2012) by both storyteller and recipients, (ii) averted gaze by storyteller (Kendon, 1967; Auer, 2018), (iii) use of person and place references (Dingemanse et al., 2017) by storyteller, (iv) shifting from present tense to past tense (Rossano, 2012), all at sequence openings. Further (v) the suspension of ordinary turn-taking in favor of the storyteller in terms of turn order, turn size, and turn distribution (Sacks, 1992; Rühlemann, 2013; Rühlemann and Gries, 2015) facilitating the storyteller’s ‘control’ of “a third slot in talk, from a first” (Sacks, 1992: 18), a pattern referred to as the ‘N-notN-N pattern’ (Rühlemann and Gries, 2015), (vi) the concomitant reduction of story recipients’ contributions to producing continuers (Schegloff, 1982) acknowledging the “structural asymmetry” (Stivers, 2008: 34) of the telling sequence and, closer to the story highpoint, affiliative tokens (Stivers, 2008) mirroring the storyteller’s stance toward the events (Stivers, 2008: 33), (vii) the Labovian a-then-b event structure (Labov and Waletzky, 1967; Labov, 1972), (viii) the sequential organization of talk “in larger structures” (Goodwin, 1984) including Preface, Background, and Climax, (ix) heightened occurrence of constructed dialog (Labov, 1972; Mayes, 1990; Holt, 2000), as well as (x) sequence-final pausing (Rühlemann et al., 2013), aversion of mutual gaze (Auer, 2018), and sequence-recompletion (Hoey, 2017).

Climax identification too was based on a set of criteria including the following: increased occurrences of (i) ‘narrative clauses,’ that is, clauses bracketed by a temporal juncture (Labov and Waletzky, 1967: 27–28; Labov, 1972: 361) often marked syntactically by being independent clauses and containing simple past and simple present verb forms (Labov, 1972: 364), (ii) intensified use of ‘stance devices’ (Stivers, 2008; cf. Labov’s [1972] ‘evaluative devices’) such as, most prominently, constructed dialog (Labov, 1972; Longacre, 1983; Li, 1986; Mayes, 1990; Holt, 2000) often co-occurring with storyteller’s gaze aversion (Sidnell, 2006) and produced potentially also by recipients (Rühlemann, 2013), (iii) increased production by story recipients of high-involvement (affiliative) response tokens (Stivers, 2008) including laughter (Mandelbaum, 2013: 499) and (non-minimal) assessment tokens (Goodwin, 1986; McCarthy, 2003), and (iv) increased levels of storyteller’s multimodal behavior (Blackwell et al., 2015; Stec et al., 2016).

The data selected for this paper comprise two storytellings that are similar and dissimilar with regard to a number of key properties. They are similar in that they are both triadic, same-sex, slightly over a minute long (66 s for “Toilet woman,” 75 s for “Sad story”), feature a single storyteller, contain co-climactic assessment tokens by the story recipients, and, importantly, they centrally convey emotions of the same emotion family, namely sadness/distress.^4^ They are dissimilar in that the storyteller’s stance is conveyed verbally in “Sad story” but multimodally in “Toilet woman”; they differ in terms of participation framework (recipient B in “Sad story” is storyteller C’s brother and hence the same ‘party’), “Sad story” is a third-person story, while “Toilet woman” is first-person, and, importantly, initial inspection of the video-recordings suggested high levels of multimodal investment in “Toilet woman” but low levels in “Sad story.” The two stories thus stake out the *range of multimodal storytelling performance* and (some of) their contingencies to be expected in a larger data collection and thus allow for an initial assessment of what factors may facilitate or impede emotion contagion.

### Electrodermal activity

The participants to this study wear Empatica wrist watches taking a variety of psychophysiological measurements, including EDA. The wrist watches’ sampling frequency for EDA measurements is 4 Hz within a range of 0.01–100 μSiemens. With the watches being placed at the wrists, EDA is measured in close proximity to the palms where the highest concentration of eccrine sweat glands is found (as opposed to apocrine sweat glands, for example, in armpits and genital areas). While the primary function of most eccrine sweat glands is thermoregulation, it has been suggested that eccrine sweat glands on and near the palms are involved in “emotion-evoked sweating” (Dawson et al., 2000: 202) rather than “physical activity or temperature” (Bailey, 2017: 3). Given its strong association with emotion, EDA can count as a physiological symptom of emotion (Scherer, 2005; Bradley and Lang, 2007)^5^. Unlike other emotion-related measures resulting from muscular activity and cardiovascular activity (such as heart rate and blood flow), which are ambiguous indices of arousal, EDA is a more reliable indicator of emotional *arousal* (Peräkylä et al., 2015). Arousal represents the intensifying excitation of the sympathetic nervous system associated with emotion (Dael et al., 2013: 644; Peräkylä et al., 2015: 302). EDA reflects changes in sweat gland activity and skin conductance: emotional excitement correlates with increases in EDA while emotional calmness correlates with decreases in EDA. Being controled by the sympathetic nervous system either process is beyond volitional influence.

A distinction is commonly made between tonic and phasic EDA, the former referring to “the average level of skin conductance, resistance or potential in a given situation” (Lykken and Venables, 1971: 657) and the latter referring to “transient, wave-like changes which may be elicited by external stimuli or may be ‘spontaneous,’ i.e., elicited by internal events” (Lykken and Venables, 1971: 657).^6^ In the present research, the focus is on phasic EDA, as, based on the notion of emotion contagion, the prediction is that the storyteller and the recipients will exhibit *roughly synchronous EDA responses* at a short predefined interval, namely the Climax, with the storyteller’s EDA response elicited by the internal stimulus of reliving of the events depicted at Climax, and the recipients’ response elicited by the storyteller’s telling about the climactic event. The predicted synchrony at Climax is expected to be moderated by onset latency, which is typically 1–3 s (Dawson et al., 2000: 206). Another possibly moderating (or even confounding) factor is the substantial inter-subjective variability in EDA responsiveness, which may be due to physiological or psychopathological conditions (Lykken and Venables, 1971; Dawson et al., 2000). Within these limits of inter-individual differences, the intensity of an EDA response and, hence, the intensity of emotional arousal can be read off the responses’s *amplitude*, with large amplitude increases indicating high-intensity arousal (Bailey, 2017: 8).

### Multimodal methods

As noted earlier, quotations are exhaustively annotated during transcription in ELAN. Given their annotation on a separate tier they not only have exact temporal properties but can be automatically retrieved. The annotation of quotation also includes so-called silent quotes, that is, depictions of non-verbal behavior that do not occur co-speech (cf. Hsu et al., 2021).

The two storytellings under scrutiny were subjected to prosodic analysis in Praat, a widely used phonetic analysis tool (Boersma and Weenink, 2021). A script written by David Weenik helped extract from the storytellings *synchronized* intensity and pitch values. Scatter plots with overlaid locally weighted regression lines were used to analyze the coincidence, or lack thereof, of pitch and intensity peaks with the respective story Climaxes.

The participants wear Ergoneers eye tracking glasses recording their foveal vision, that is, the field of vision with greatest visual acuity (e.g., Auer, 2021). A distinguishing feature of the Ergoneers eye trackers is that researchers have access to the eye camera videos. The benefit of this accessibility is that missing or erroneous pupil detections can be manually corrected. As this manual correction is done frame-by-frame it is time-consuming and cannot be afforded for large-scale analyses. However, for the present study, in which selected storytellings were analyzed, pupil detection was manually corrected.

Further, gaze fixations were manually annotated in ELAN, with a focus on five major gaze directions:

– to each face of the two co-participants,
– between the two co-participants,
– to the side,
– up,
– down.

Finally, any participant’s foveal vision is continuously tracked and recorded as X/Y coordinates in the participant’s field of vision. This allows the researcher to trace gaze movements across time. Of particular interest in this study is whether the storyteller’s gaze shows any movement pattern(s) in relation to the telling’s progression toward the Climax.

**FIGURE 1:**
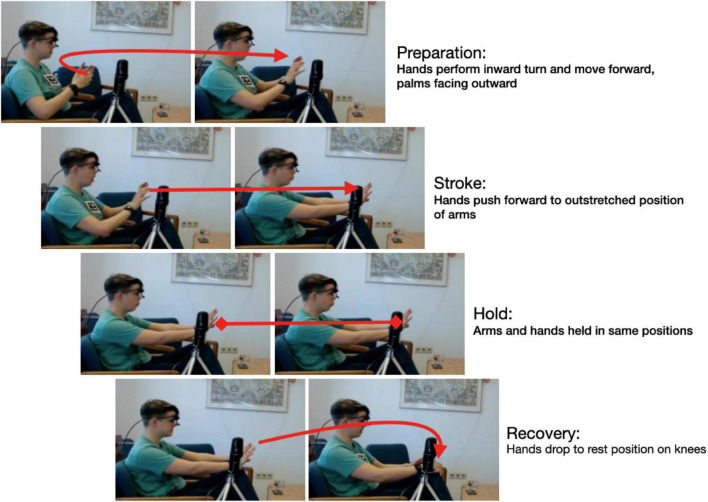
Gesture phases illustrated: a *gesture unit* featuring all four gesture phases.

Gesture annotation was implemented manually in ELAN. Gestures were annotated on two planes: gestures phases and gesture dynamics. Gesture phase annotation was based on Kendon’s (2004) gesture phase model. It distinguishes four phases: preparation, i.e., the departure of the hand(s) from a rest, or home, position; the stroke, i.e., the “phase of the excursion in which the movement dynamics of ‘effort’ and ‘shape’ are manifested with greatest clarity” (Kendon, 2004: 112); the (optional) hold, i.e., the “phase in which the articulator is sustained in the position at which it arrived at the end of the stroke” (Kendon, 2004: 112); and the recovery, i.e., the movement from the stroke (and optional hold) back to the home position. Stroke and hold together form the nucleus, which is that part of the gesture “that carries the expression or the meaning of the gesture” (Kendon, 2004: 112). It is not unusual for speakers to use gesture sequences without intermittent return to home position. Where this is the case, the individual gestures consist of gesture phrases only whereas a gesture that does return to home position counts as a gesture unit. The phases are illustrated in Figure 1:

The way gestures and gesture phases are annotated in ELAN is on two separate tiers, one for the gesture as a whole and one for the phases. This two-tier annotation enables the direct generation of exact durations of the whole gesture as well as its distinct phases. Moreover, the gesture description also records the articulating organ(s) involved: the initials ‘m,’ ‘f,’ ‘h,’ and ‘t’ are used for hand gesture, facial expression, head gesture, and, respectively, movement of the torso including the shoulders. To illustrate, consider the gesture description for the gesture depicted in Figure 1:

(m & t & f: in sudden move, torso slightly bent forward, eyes and mouth wide open, both hands stretch out, palms facing outward).

The gesture description makes it clear that this is a multi-articulator gesture with the involvement not only of the hands and arms but also the torso and the face (eyes and mouth, in this case).

Gestures are furthermore annotated in ELAN for gesture dynamics (e.g., Dael et al., 2013). A number of recent studies have shown that dynamic qualities of body movement add to the movement’s expressive potential and carry emotionally relevant information (e.g., Atkinson et al., 2007; cf. Dael et al., 2013 and references therein). To capture gesture expressivity we examine gestures based on what we refer to as Gesture Expressivity Index (GEI). This index comprises five variables, with the value 1 assigned if TRUE and 0 if FALSE. Underlying the variables is Dael et al.’s (2013: 653) finding that increased emotional arousal results in more abundant movement. Unlike Dael et al. (2013), however, who presented raters with six continuous variables (amount, speed, force, size, fluency, and height)^7^, the aim in the present study was to reduce the number of continuous variables and instead give weight to strictly binary categories that are *per se* rather unambiguous in that they are immediately available or computable either from the ELAN gesture phase annotation or directly accessible from inspection of the video-recordings. The variables used include three strictly binary variables as well as two continuous variables. The binary variables are as follows:

*ND: Nucleus duration is greater than story-average.*

As noted, the nucleus, as the combination of stroke and optional hold, “carries the expression or the meaning of the gesture” (Kendon, 2004: 112). We assume that if that expression or meaning is actively displayed to the interlocutor in a prolonged manner, the interlocutor’s perception of the expression or meaning will be facilitated. As a reference measure, the gesture’s nucleus duration is compared to the mean nucleus duration in the storytelling. The value 1 is obtained if the duration is greater than the mean nucleus duration.

*SL: Gesture is silent gesture.*

Silent gestures, alternatively referred to as ‘speech-embedded non-verbal depictions’ (Hsu et al., 2021), are gestures that communicate meaning “iconically, non-verbally, and without simultaneously co-occurring speech” (Hsu et al., 2021: 1). With the (default) verbal channel muted, the burden of information is completely shifted to bodily conduct (cf. Levinson and Holler, 2014: 1). This shift makes silent gestures particularly expressive: they are ‘foregrounded’ and ‘exhibited’ (Kendon, 2004: 147). Actively attending to them is prerequisite for the recipient’s understanding. Moreover, given that the occurrence of speech is expected, its absence will not only be noticeable but also emotionally relevant as the omission of an expected stimulus has been shown to increase EDA response (Siddle, 1991: 247).

*MA: Gesture is performed by multiple articulators.*

This variable is readily available from the ELAN gesture annotation, where, as noted above, the description of the gesture is preceded by the initial of the articulating organ(s). If there is a single initial, the hand movement is the only visible bodily conduct; if there is more than one initial the gesture is performed in synchrony with other bodily articulators, e.g., the head, the face, the torso, and, in some cases, the eyes (see below). The rationale for inclusion of this variable in the Index is again Dael et al.’s (2013) above cited finding that arousal is positively correlated with amount of bodily movement. This variable also allows us to take facial expression into account.^8^

The gestures were also rated for two continuous variables, namely *gesture size* (SZ; Gesture is expansive) and *gesture force* (FO; Gesture requires muscular effort), two of the parameters familiar from Dael et al.’s (2013) analysis. To ensure comparability, size and force were equally coded in a binary fashion. Being continuous, these dynamics are not easily captured on a binary scale and likely subject to rater disagreement. Therefore, the two criteria as well as all other criteria were rated by two raters and rating agreement percentages were calculated.

The Index is computed for each gesture as the average value on all five variables. Given that variable values are either 1 or 0, GEI values range between 0 and 1, with values close to 1 signifying substantial expressivity and values close to 0 signifying weak expressivity. To establish the overall expressivity across the storytelling as a whole, a grand mean is computed (termed mean GEI). To obtain a metric that indexes the gestures’ *expressivity development* across the storytelling, a linear regression is calculated based on the Index values of all gestures occurring prior to the Climax (thus excluding gestures and their GEI values occurring in mid-Climax or post-Climax position). Crucially, expressivity development is inferred from the *slope* of the regression: a positive slope value is seen as indexing intensifying expressivity toward the Climax; small or negative values indicate the gestures become less expressive as the telling approaches its Climax.

## Material

In this section, the two storytellings examined are presented.

### “Toilet woman”

The first story “Toilet woman” is given in extract (1). The storyteller, speaker A, is relating the distress caused by the fact that she is frequently mistaken for a man and thus denied basic services and facilities such as the use of public bathrooms for women. Prior to the extract speaker A has described various situations where using a public bathroom proved problematic as people mistook her for a man. The story in extract (1) is introduced in line 1 as a description of “[like] no:rmally how it happens,” which frames the upcoming story as a typical incident and also acts as a story preface (Sacks, 1992). The lengthy Background section starts with the storyteller ascertaining there is common ground regarding the particular bathroom in the train station (lines 1–21). The Background continues with two temporally separated events connected by the fact that on both occasions her gender was not recognized by the toilet women; in the first incident (lines 23–29), she was first denied entry into the women’s toilet until the toilet lady recognized her as a woman. In the second incident (lines 30–44), occurring “a week later,” she is again denied entry by another cleaning lady, which prompts an incredulous reaction from A expressed in the silent gesture “I’m like ∼(silent f: blank stare)∼.” As a result, a kind of showdown develops: “I just !look!ed at her and >she was also< much shorter than me so <I’m like looking down at her>.” At that critical moment the cleaning lady “from the week be↑fore↑ who had ↑already↑ done this” intervenes in A’s favor. Her constructed dialog delivered in high voice “∼↑!THAT’S! A WOMAN (.) [!THAT’S! A WOMAN↑∼” (line 45) represents the first part of the Climax. Although this declaration resolves the misunderstanding, the emotions displayed in A’s silent gesture: “I’m like ∼(silent m and f: drops hands from arm rest, pulls corners of mouth)∼” (lines 46–47), which concludes the Climax and is shown in figure 1 in extract (1), express frustration and exhaustion, reflecting the discrepancy that a simple visit to a public bathroom turns into a predicament, and, for her, does so on a “normal” basis. Note that the recipients seem to empathize with A’s predicament: already upon learning that the toilet woman from the week before intervened (i.e., immediately before the Climax) speaker B displays her sympathy with A (as well as her amusement over the funnier details of the story) by uttering “£°aw°£” in a low smiley voice (line 42), while speaker B responds after the Climax with a lengthened “=holy [sh:]i:[:t” and extends the sequence with the concerned question “does it happen] often? [like]” (line 50).

Note further that the story is told linearly, i.e., the temporal chain of events is continuous, leading from the first event (the first visit to the bathroom) to the event depicted in the Climax (the second visit a week later) in a straight progression.

**Table:** 

(1)		FreMIC F01[208–252]
01	A:	= [like] no:rmally how it happens
02		so it’s happened <in the> Hauptbahnhof a lot,
03		(0.804)
04	A:	because like tho:s:e
05		(0.480)
06	A:	I don’t know if you have ever used that bathroom,
07		in the train station¿=
08	C:	=[no]
09	B:	=[no]
10	A:	it’s (.) ↑!it! is↑ kind o:f: you ha-
11		it’s not intuitive which one’s the men’s
12		which one’s the women’s like you need to really
13		like<read the si:[gn]>
14	C:	[oh the one] you have to pay to
15		[enter¿ like one]
16	A	[!yeah!]
17	C:	euro [or something] °yeah yeah°
18	A:	[yeah =yeah =yeah =yeah]
19	A:	and like IT’S AN OPEN entrance on either end (.)
20		I get it you go in the wrong end
21		(0.211)
22		u:m (.)
23		I was going in one day and the cleaning woman there
24		!stopp!ed me >she was like<
25		(0.384)
26	A:	∼er (0.237) sorry this is the women’s∼
27		and I turned around she goes (.)
28		∼(v: gasps) I’M SO SORRY >>go in go in go in go in<<∼
29		(0.331)
30	A:	and like a!week! later the same bathroom going in the:re
31		(0.394)
32	A:	and an!other! woman!stop!ped me
33		like she’s coming out the stall and she’s like
34		∼(v: gasps) WAIT (.) THIS IS THE WOMEN’S ’∼ (.)
35		and I’m like ∼(silent f: blank stare)∼
36		(0.850)
37	A:	and I just !look!ed at her and
38		>she was also< much much shorter than me so
39		<I’m like looking down at her>
40		and the ↑cleaning↑ woman is there
41		from the week be↑fore↑ who had ↑already↑ done this=
42	B:	= £°aw°£ =
43	A:	= >and she went< !out of her way! to come in > and be like<
44		(0.280)
45	A:	∼↑!THAT’S! A WOMAN (.) [!THAT’S! A WOMAN↑]∼
46		I’m like# ∼%(silent m and f:
47		drops hands from arm rest, pulls corners of mouth%)∼
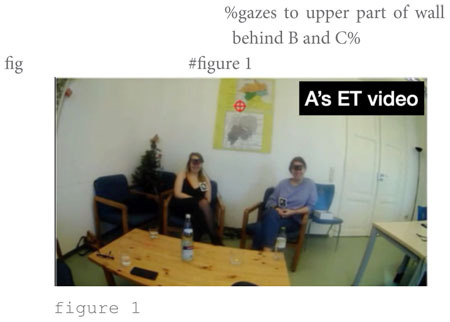		
48	B:	[(v: laughs)]=
49	C:	[(v: laughs)]
50	C:	=holy [sh:]i:[:t does it happen] often? [like]
51	A:	[jus:t] [gonna (.) stand here] [yeah]

### “Sad story”

The second story is entitled “Sad story” because the storyteller, speaker C, introduces the story explicitly as a “story” and bills it as a “*sad* story” in line 1: “BUT THAT was (.) a sa:d story.” This is noteworthy as the speaker here explicitly names the emotion the story is supposed to convey; more commonly, story prefaces, such as this one, implicitly betray the storyteller’s stance toward the events (Stivers, 2008). B goes on to add “(if) we got the time°f- fo(h)r it [here]°” (lines 2–3) with a quick look to speaker B (in the middle of the room camera feed) who we learn earlier in the recording is his elder brother. His brother’s “[oh] yeah” and the accompanying laughter (line 4) give him the green light to tell the story.

**FIGURE 2:**
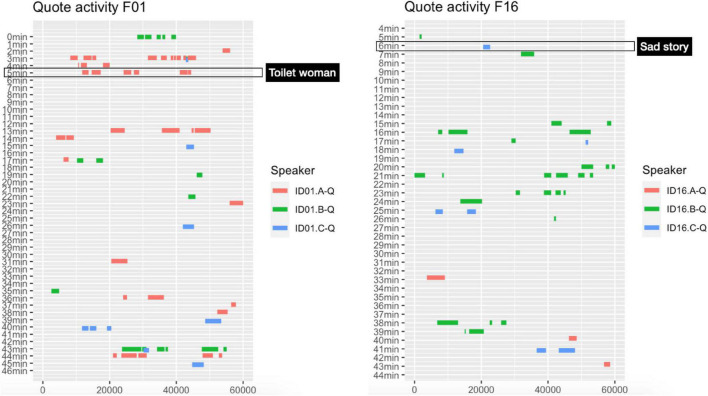
Quote activity in the two files F01 and F16 from which “Toilet woman” and “Sad story” were excerpted.

The focus of the story are their father’s early career struggles. He embarked on a career as a diplomat (line 7) and took the first steps (lines 8–11), which B evaluates as “[!really! h]ard like I’m I’m not even attempting it.” That external evaluation (Labov, 1972) is taken up by speaker A, who compares the father’s arduous training in the diplomatic sector to joining “the foreign] service, [right¿] [I think it takes like] three years [doesn’t it]°to [do everything°]” (lines 13–21). This lengthy intervention can be seen as ‘disruptive’ (Mandelbaum, 2013: 502) to the storyteller’s storytelling project; at least, after resuming the telling (line 24), C runs into serious trouble searching for a near-synonym for “budget deficit” (lines 25–34). During the lengthy word search he avoids looking at the recipients but looks down to the table in front of him and to its side (s. figure 1 in the transcript). Only when speaker A finally provides the missing word “fiscal cliff” (line 35) does he look up again, acknowledges the term with “yeah] °yeah°” (line 37) and resumes the telling by relating that, as a consequence of the fiscal cliff, “all new positions are cut” (line 39) including his father’s position (line 41). The Climax is arguably reached when C reports that “they tell him ∼you have to reapply and try get in again∼” (lines 43–44), stressing that it is “!super! hard to get in and [pass these] tests” (lines 45–46). At this point, C inserts that “like our oldest brother >was already born<” (line 50) and “Alex the second oldest was already on the way°and so like°°needed the money°°” (lines 53–54). This post-Climax insertion goes some way toward serving as what Labov (1972) called ‘external evaluation’ where the storyteller “stop[s] the narrative, turn[s] to the listener, and tell[s] him what the point is” (Labov, 1972: 371). While ‘the point’ is not explicitly stated, the listener need not work hard to infer that losing your job when you need to feed two kids is tough or, perhaps, ‘sad,’ as was adumbrated in the story preface. Note that in other storytellings, information that is key for the recipient to grasp the point of the story is often not provided toward the end of the story, as an afterthought, but more commonly, and arguably more effectively, early in the story as part of the Background, that is, as an element of the *ground* against which the *figure* of the point of the story can be appreciated. This storytelling technique is termed ‘foreshadowing’; it refers to a process by which the “narrator knows what will follow and casts characters and events in terms of this future trajectory” (Ochs and Capps, 2001: 5). Here, then, the storyteller fails to foreshadow but provides Background information in post-Climax position. The reversal of the telling trajectory at that point breaks the linearity of the telling progression. That this post-Climax information is indeed *delayed* information transpires from speaker B’s conduct: as shown in figure 2 in the transcript, he pours himself a drink when his brother provides the information, thus disattending from the telling and treating it as completed.

Note finally that the father’s distress is mirrored by the recipients. Upon learning at the start of the Climax that “his position as a as a diplomat is cut” (line 41), recipient A utters “wow” (line 42), a ‘typical’ affiliation, or assessment, token (Goodwin, 1986; Stivers, 2008) that, here, recognizes the magnitude of the hardship involved. Similarly, recipient B’s protracted “ye:ah (.) ya” (line 47), uttered with lowered head in response to the Climax, recognizes and affirms the extreme difficulty of the situation.

**Table:** 

(2)		FreMIC F16[22677 - 22714]
01	C:	BUT THAT was (.) a sa:d story I mean
02		(if) we got the time
03		°f- fo(h)r it [here]°
04	B:	[oh] yeah [(v: laughs)] (v: laughs)
05		(0.356)
06		[u:m]
07	C:	like (.) he applied, to become a diplomat
08		(0.460)
07	B:	[yeah]
08	C:	[!got!] accepted starts with diplomatic (.) schooling
09		like he goes through the whole thing which is [!really! h]ard
10	A:	[right]
11	C:	like I’m I’m not even attempting it
12		(0.767)
13	A:	RIGHT this is [like to join the foreign] service, =
14	C:	[cos I think you sh-]
15	A:	=[ right¿]=
16	C:	[yeah ex]actly =
17	A:	[I think it takes like] three years=
18	B:	[yeah]
19	A:	=[doesn’t it]=
20	B:	[yeah]=
21	A:	=°to [do everything°]=
22	C:	=[°yeah°]=
23	B:	=°yeah°=
24	C:	= u:m and then they had a ↑budget cut. (.)
25		≈um oh I mean u:h:≈ (0.526) the United States reaches its# um
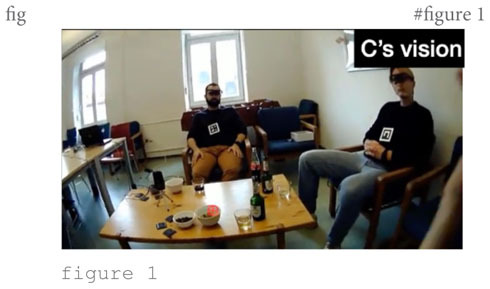		
26		(1.127)
27		budget deficit,
28		>its maximum budget deficit< °what’s° (.)
29		°what’s that called again?°
30		(0.678)
31		°governmental°
32		(0.835)
33		tt >I don’t know so there’s a government shutdown<
34		(0.297)
35	A:	the [fiscal cliff]
36	C:	[a:nd the!fis]cal!=
37		=[yeah °yeah° (.)] and (.) uh=
38	A:	[°°yeah°°°yeah°]
39		C. =and so as a result all new positions are cut (0.545)
40	A:	[mm]
41	C:	[uh and] his position as a as a diplomat is cut (0.225)
42	A:	w[ow]
43	C:	[uh an]d they tell him
44		∼you have to reapply and try get in again∼
45		which is (.) I me- (.)!super! hard to get in and
46		[pass these] tests =
47	B:	[ye:ah (.) ya]
48	C:	= [and] (.) everything (0.317)
49	B:	[°yeah°]
50	C:	and so he was already like our oldest brother > was already born <
51	B:	y[eah]
52	C:	[u:m]#
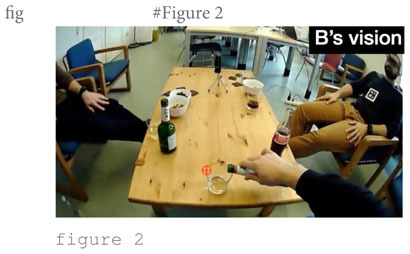		
53	C:	and I think (.) Alex the second oldest was already on the way
54		°and so like°°needed the money° °and went to uh° (.)
55		to become a lawyer for the military
56	A:	.tch °°oh°° for the for the United States military=
57	C:	=yeah=

The following section presents the results of the analyses of the storytellings. It starts with examination of EDA in all six participants focusing on their amplitudes during Climax and degrees of synchronicity between teller and recipients, continues with the results of the analyses of the storyteller’s multimodal choices, over which they exert control, and ends on consideration of factors lying outside their control such as participation framework and relevance.

## Results

### Electrodermal activity

**FIGURE 3:**
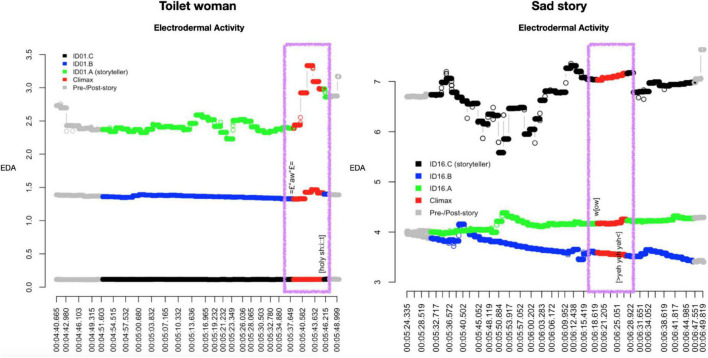
Scatter plots of EDA responses by participants in storytelling “Toilet woman” **(left panel)** and storytelling “Sad story” **(right panel)**.

Analysis of the electrodermal responses by all participants in the two storytellings, shown in Figure 3, allows for several observations.

First, the overall levels of EDA are quite distinct for all six subjects. This may be a reflection of the large inter-individual variability that exists, for example, due to the thickness of the corneum, the extreme outer layer of the skin but also to individual electrodermal ‘lability’ (Dawson et al., 2000: 209).^9^

Second, in both storytellings, the overall response is notably higher for the storytellers than the story recipients. This is to be expected because the motor and breathing activity of the storytellers is greater than that of the story recipients, who do much less talking and gesticulating.

Third, the electrodermal responses by the two storytellers at Climax are similar in kind but distinct in amplitude. For storyteller A, as shown in Table 1, EDA sees a sudden jump from roughly 2.289 μSiemens at Climax beginning to 3.332 μ Siemens during Climax (an increase by almost 40%!). This is a significant amplitude increase indexing high-intensity arousal. For storyteller C, by contrast, the maximum electrodermal response occurs far prior to the Climax (at timestamp 5:53.917)—coinciding, it turns out upon inspection, exactly with the onset of the word search—that is, more than 20 s before the highpoint is reached (at 6:15.419). For him, then, the most arousing stimulus is not the arrival at the Climax but the struggle to retrieve the term ‘fiscal cliff.’ At Climax, there is a small electrodermal response by storyteller C: the amplitude shifts from 7.031 μSiemens at Climax beginning to 7.159 μSiemens during Climax, an increase by 0.128 μSiemens. This increase is large enough to indicate a specific (i.e., stimulus-related) rather than non-specific (i.e., stimulus-unrelated) response, for which a threshold of 0.05 μSiemens is commonly used (cf. Dawson et al., 2000: 206). However, the increase in storyteller C’s response is vanishingly small compared to the increase in storyteller A’s response, indexing low-intensity arousal.

**TABLE 1:** Electrodermal activity (EDA) during Climax by participants and stories.

	Toilet woman			Sad story		
Role	Recipient	Recipient	Storyteller	Storyteller	Recipient	Recipient
*Participant*	*C*	*B*	*A*	*C*	*B*	*A*
EDA at Climax beginning	0.114	1.326	2.389	7.031	3.524	4.250
Maximum EDA during Climax	0.118	1.469	3.332	7.159	3.589	4.164
Amplitude change	0.004	0.143	0.943	0.128	0.065	−0.086

**FIGURE 4:**
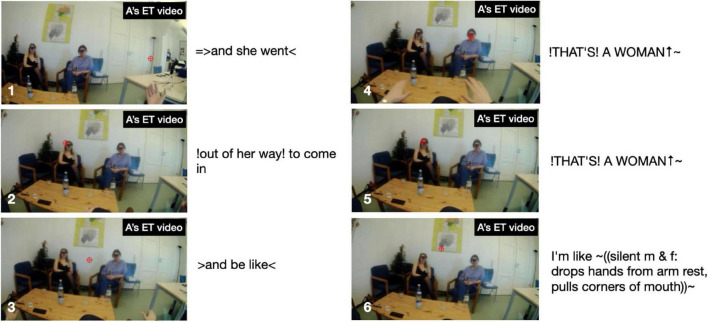
Storyteller’s gaze locations during the Climax in “Toilet woman”.

Fourth, in both storytellings, the recipients’ electrodermal responses partly trend in different directions. For recipient B in “Toilet woman” there is a clear hike in EDA during the Climax and, what is more, it occurs in near-perfect synchrony with the storyteller’s spike in EDA. The two spikes occur when the storyteller says in loud and high-pitched voice “↑!THAT’S! A WOMAN (.) [!THAT’S! A WOMAN↑]” (line 45 in extract 1) while twice performing a “no” gesture with both hands forcefully tracing the shape of a big X (from shoulders to knees), thus reenacting the first toilet lady’s intervention on her behalf (cf. the stills in Figure 4 below). Recipient B’s response constitutes a ‘specific’ (i.e., stimulus-related) response, as the step-up from 1.326 μSiemens at Climax beginning to 1.465 μSiemens during Climax is 0.139, far in excess of the 0.05 threshold for non-specific responses. In other words, the electrodermal responses by the storyteller and B are fully aligned. Note also that, as can be seen in Figure 3, B’s maximum amplitude is reached with a 1.5 s delay compared to A’s maximum amplitude; this onset delay is squarely within the typical range of onset delays and is thus further evidence that B’s response is a response to the storyteller’s stimulus, the Climax. Here, then, we may observe emotional contagion: storyteller A’s emotion of distress resonates in recipient B. To the extent that, following Ekman (2014), we view emotional resonance as indexing *empathy*, we can say that recipient B empathizes with storyteller A. What the EDA evidence does not tell us is whether that potential empathizing constitutes a (altruistic) sympathic reaction or a (egoistic) personal distress reaction. By contrast, in the same storytelling, recipient C’s response remains virtually flat throughout, as can be seen from Figure 3, except for the slight amplitude change during Climax of 0.004 μSiemens, which falls below the threshold for non-specific responses; in other words, for this participant we see no signs of emotional arousal or contagion anywhere in the telling including the Climax.^10^

**FIGURE 5:**
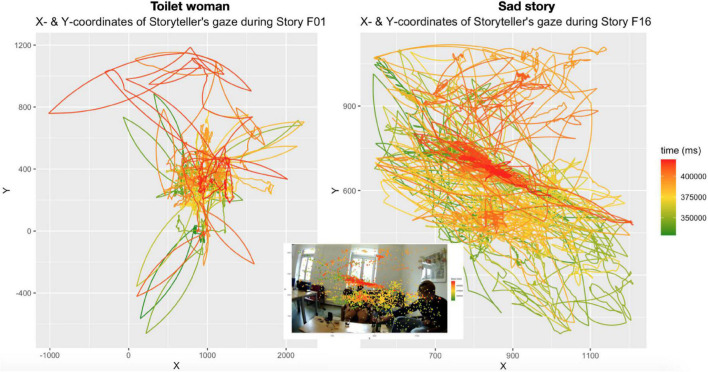
Gaze movements by storytellers based on continuous color scale aligned to time: from green (story-early) to gold (mid-story) to red (story-final); Inset: gaze scatterplot overlaying still from storyteller C’s eye tracking video.

We observe similarly different trends for the recipients’ responses in “Sad story”: recipient A, the main addressee, does exhibit the expected EDA response during Climax—however, only minimally, with a step-up in amplitude of 0.065 μSiemens but also with the expected onset delay. The peak occurs when storyteller C stresses the hardship involved in “passing these tests” (lines 46 in extract 2) said simultaneously with the preparation of an open-palm lateral sweep of the right hand on “and everything” (line 48). Recipient B, by contrast, the secondary addressee, does not show any response but his EDA continues its overall downward trend during Climax, resulting in a negative amplitude change of −0.086. That is, as with “Toilet woman,” we may see emotional contagion in “Sad story” in one of the two recipients, recipient A; however, the contagion load is far less substantial than in “Toilet woman.”

Fifth, the absence of EDA responses and, hence, emotional resonance at Climax in two of four recipients is at odds with the fact that that *all* recipients produce engaged response tokens around the Climax: in “Toilet woman,” recipient B produces a sympathic “£°aw°£” softly spoken and with smiley voice, and even recipient C, whose EDA remains noteably unchanged during the storytelling as a whole, displays empathy using the elongated expletive “holy sh:i:t”; in “Sad story,” recipient A, who does respond electrodermally at Climax, utters “wow,” an assessment/affiliation token (Goodwin, 1986; Stivers, 2008) whereas recipient B, who shows a decrease in responsivity during Climax, produces an empathic-sounding “ye:ah (.) ya” spoken with lowered head.

The fact that both recipient B’s “£°aw°£” in “Toilet woman” and recipient A’s “wow” in “Sad story” occur *prior* to the respective Climaxes need not surprise us given that, according to emotion construction theory, recipients may predict what emotion the storyteller will express based on their own past experiences and on the ‘traces’ laid out in the storytelling so far. Considering that the two recipients in question are at the same time those who emotionally resonate with the storyteller, it is tempting—but, given the scarcity of data, highly speculative—to entertain the possibility that such ‘early’ empathy displays index a higher degree of allostatic prediction work and will therefore positively correlate with achieved resonance. Though speculative at this point, this possibility presents an intriguing avenue for future research.

The results of the EDA analyses are uncomfortably mixed: there is a little bit of everything. While we get a consistent set of empathic emotion displays by all four recipients of the kind expected in stories of distress, that consistency is not matched by the participants’ EDA responses. We see upticks in EDA response in both storytellers at Climax and synchronous EDA upticks in one recipient in each story. But the magnitudes of these responses point in opposite directions: they are large in “Toilet woman” and small in “Sad story” suggesting there is more emotional resonance in “Toilet woman” than in “Sad story.” On the other hand, recipient B’s EDA response in “Sad story” shows a downward trend at Climax and recipient C’s response in “Toilet woman” stagnates completely.

How can this variance be explained? One obvious explanation is the inter-individual variability inherent in how emotions are constructed based on concepts compressed from past experiences (cf. Introduction). As we have no evidence of these processes we need to turn to other forms of evidence for explanation. The remainder of the Results section provides and discusses ample evidence gained from a wide range of methods. I will start with the multimodal analyses focusing on the storytellers’ verbal and non-verbal behavior before I consider factors lying outside of the storyteller’s control.

### Under the storyteller’s control: Multimodal analysis

#### Quotation

Figure 2 shows quote activity for the two video-recordings from which the two storytellings were extracted. It can be seen that over the course of the whole recording, speaker A (indicated as ID01.A-Q) uses quotation quite frequently, whereas speaker C (indicated as ID16.C-Q in the figure) uses just a handful. During the storytellings under examination that discrepancy holds true too: while speaker A uses a total of six quotations, speaker C uses only one.

As a consequence of the much higher number of quotes in “Toilet woman,” much more time is spent doing quotation: speaker A’s quotes have a 17.9% share in the total telling time, whereas speaker C’s lone quote merely accounts for 2.8%.

But the quotes by the two storytellers do not only differ in number but also qualitatively. There are for speaker A two *silent* gestures used as quotations: “∼(silent f: blank stare)∼” and “∼(silent m and f: drops hands from arm rest, pulls corners of mouth)∼.” Such *silent quotes*—a subtype of ‘speech-embedded non-verbal depictions’ (Hsu et al., 2021)—can be seen as particularly expressive for two reasons: speech is halted, resulting in silence, and, instead of descriptive speech, a gesture (iconically) depicts in the here-and-now a meaning that is referenced to a different spatio-temporal setting. Also, the second silent quote, a screenshot of which is shown in figure 1 in transcript (1), occurs at a key location as it concludes the Climax.^11^

Further, we see differences in the vocal design of the *non*-silent quotes. While the transcription suggests no changes in paralinguistic prosody in speaker C’s quote “∼you have to reapply and try and get in again∼,” a wealth of indications of paralinguistic variability is found in the transcriptions of speaker A’s quotes, including gasping, pausing, acceleration (indicated in < … >) high pitch (indicated in ↑), and loud voice (indicated by upper-case letters):

"∼er (0.246) sorry this is the women’s∼"
"∼(v: gasps) I’M SO SORRY > go in go in go in go in < ∼"
"∼(v: gasps) WAIT (.) THIS IS THE WOMEN’S∼"
"∼(silent f: blank stare)∼"
"∼↑!THAT’S! A WOMAN (.) [!THAT’S! A WOMAN↑∼"
"∼(silent m and f: drops hands from arm rest, pulls corners of mouth)∼"

So a first reason why the emotional resonance in “Toilet woman” is larger may be due to the higher frequency and the much more variable prosodic design of quotation by storyteller A in “Toilet woman.”

#### Prosody: Pitch and intensity

**FIGURE 6:**
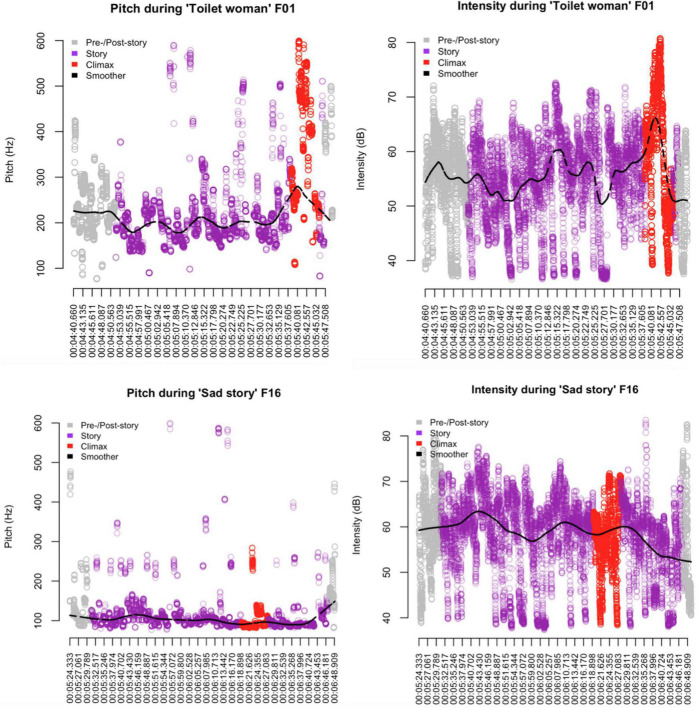
Pitch and intensity in “Toilet woman” vs. “Sad story”; smoothers (black line) represent locally weighted regression lines.

Figure 6 plots pitch and intensity for both stories; the black lines show smoothers (cf. Cleveland, 1979), i.e., locally weighted regression lines that do not impose a single model but model several small local segments of the data and that are therefore useful for detecting and comparing trends in noisy variables.

The evidence presented in the scatter plots is straightforward: while there is a great deal of variability, the smoothers for “Toilet woman” peak exactly and clearly where they are expected, namely at Climax. For “Sad story,” no such correlation is found; on the contrary, the pitch remains level in pre-Climax and Climax position and even shows a slight uptick in post-Climax position; intensity exhibits a number of humps, but none in Climax position, and the overall trend from the beginning to the end is downward.

So the greater emotional resonance in “Toilet woman” may also benefit from the storyteller’s ratching up pitch and intensity toward the Climax while the storyteller in “Sad story” does not exploit these resources to engineer emotional arousal.

To conclude this section it should also be noted that the two storytellings differ in terms of another aspect related to the verbal mode, story delivery: while “Toilet woman” was delivered with uninterrupted progressivity and in linear order, the delivery of “Sad story” was hampered by the massive word search at story beginning and a non-linear placement of crucial background information until after the Climax. Both delivery ‘flaws’ may also have contributed to the relatively lesser emotional impact of the storytelling on the recipients.

#### Gaze: Gaze fixations and gaze movements

**FIGURE 7:**
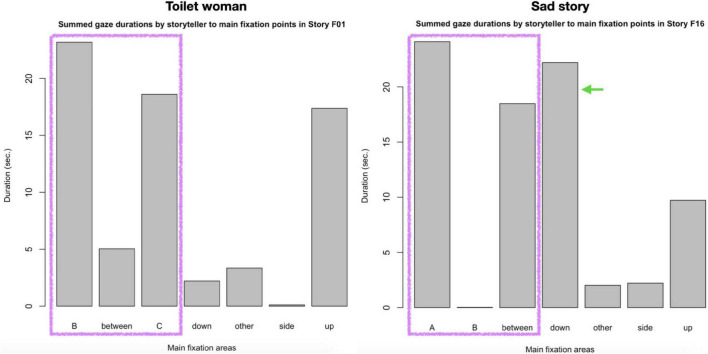
Summed durations of main gaze fixations.

The barplots in Figure 7 show the distribution of the five distinct gaze fixation points noted above: to each of the two co-participants (B and C in the case of “Toilet woman,” and A and B for “Sad story”), to a location between the co-participants, to the side, up, or down (a sixth category is other).

Storyteller A spends most of her gazes on B and C; she also notably gazes up quite a while, that is, at locations above co-participants B and C.

The contrast with storyteller C is striking: while also looking to one of the recipients (namely A) quite some time, the time spent looking at the second co-participant (namely B) is vanishingly small. Instead, the second largest time share is for gazing *down*, most of it due to C’s lengthy word search highlighted in figure 1 in transcript (2).

How do the storytellers’ gaze movements pattern as their tellings progress toward the Climax?

Figure 5 uses a continuous color range from green for story-initial points to red for story-final points in time to allow the observer to trace the storytellers’ gaze movements as the tellings unfold in time.

A first observation concerns the gaze radius, which can be read off the X/Y scales in Figure 5. While the radius of storyteller C’s gaze is relatively narrow throughout the telling, storyteller A employs a much wider gaze radius, covering a much greater field of vision in the room, including all directions (down, up, and to either side). Where Storyteller C’s gaze does gaze away too it is predominantly in one direction, namely down; notice in Figure 5 the many meandering gaze movements lower on the *Y*-axis, reflecting the gaze movements during the word search in the early half of the telling.

**FIGURE 8:**
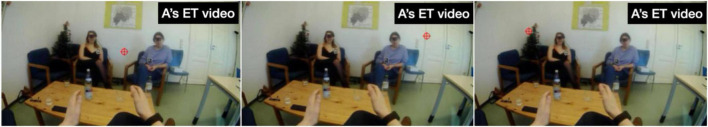
Storyteller’s gaze movements during silent quote “∼(silent f: blank stare)∼”.

Another difference relates to semantic content of gaze-away movements. Storyteller C’s looking down during a word search—an instance of a well-established behavioral pattern (e.g., Goodwin and Goodwin, 1986)—is semantically void, it does not contribute to the content of the story; rather, it is “indicative of the preference for self-repair” (Auer and Zima, 2021: 391). Storyteller A’s away-gazes, by contrast, do contribute meaning to the story. For example, when she depicts her blank stare in response to the first toilet woman denying her entrance [in the silent quote “∼(silent f: blank stare)∼”; cf. line 35 in extract (1)] she looks both to the left side of co-participant B and to the right side of co-participant C, to convey her puzzlement upon the denial, as shown in Figure 8.

**FIGURE 9:**
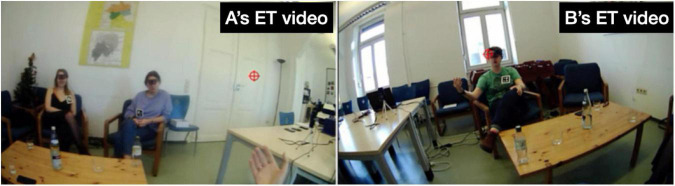
**Left panel:** Storyteller’s gaze movement to the far right end during description of the second cleaning lady’s intervention “and the ↑cleaning↑ woman is there from the week be↑fore↑ who had ↑already↑ done this=”; **right panel:** still from co-participant B’s eye-tracking video showing storyteller A’s open-palm gesture sweeping to the right in alignment with her right-sweeping gaze.

To cite another example, we observe an even farther gaze-away when storyteller A relates the intervention of the cleaning woman “from the week be↑ fore ↑.” As shown in Figure 9, her gaze shifts toward the far right end of the room, thus providing a likeness of the distance from which the intervening cleaning lady came onto the scene (note also how the gaze movement is perfectly aligned with A’s far-sweeping hand gesture to the right).

As a third example, remember from the discussion of extract (1) how during the silent quote “I’m like (silent m and f: drops hands from arm rest, pulls corners of mouth)∼,” which concludes the Climax, storyteller A gazes for an extended moment above the heads of her interlocutors, to depict her exasperation at repeatedly being mis-recognized in her gender identity. So, in “Toilet woman,” gaze radius is employed as an expressive resource to convey emotional states and, thus, muster emotion contagion. In “Sad story,” no such expressive use of gaze radius is observed.

Another observation concerns the increasing gaze *focus* on the co-participants as the telling progresses toward the Climax. This focusing process can be observed for both storytellers—but with one major difference: storyteller A includes both addressees in her ever closer focus, storyteller B focuses only on recipient A.

For illustration, Figure 4 shows six stills from storyteller A’s eye-tracking video during the Climax. At first, in still 1, on saying “ = > and she went” her gaze is at the far right end of the room, but sweeps across the room toward B on “!out of her way! to come in” (still 2), strays into the middle between B and C on introducing the quote with “ > and be like < “ (still 3), fixates on C with the first part of the quote “∼↑!THAT’S! A WOMAN↑∼” (still 4), shifts back to B for the repetition “!THAT’S! A WOMAN↑∼” (still 5), to finally center again in-between the two recipients on performing the silent quote “I’m like ∼(silent m and f: drops hands from arm rest, pulls corners of mouth)∼” (still 6). In other words, her gaze weaves a close-knit web to include and address both recipients.

**FIGURE 10:**
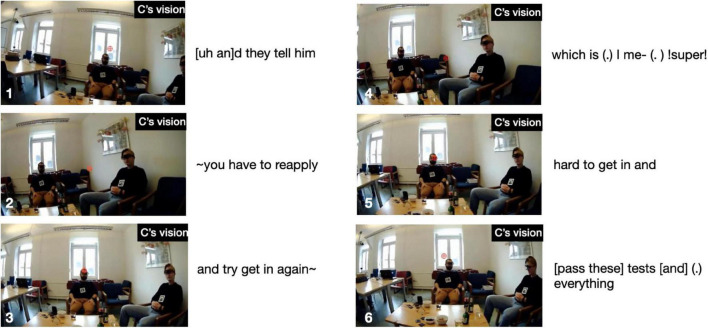
Storyteller’s gaze locations at various points during the Climax in “Sad story”.

By contrast, storyteller C’s gaze movements during the Climax are addressed virtually only to recipient A, as shown in Figure 10. In still 1, on introducing the upcoming quote with “[uh an]d they tell him,” C’s gaze focuses on A, then shifts mid-way toward B on the first half of the quote “∼you have to reapply” (still 2), switches back to A on the second half of the quote “and try get in again” (still 3), shifts back mid-way toward B on “which is (.) I me- (.)!super!” (still 4), returns to A for “!hard to get in and” (still 5), to finally focus again on A with “[pass these] tests [and] (.) everything” (still 6). So, during the Climax C’s gaze is focused on a narrow field of vision; however, that focus includes A but excludes co-participant B (this exclusive focus is also shown in the inset in Figure 5 above, where there are a few red dots sprinkled in speaker B’s direction but virtually none reaching him, while there is a massive cluster of dots across recipient A’s face).

So, both storytellers focus their gaze progressively on the co-participants during the Climax. But storyteller A does so *inclusively* by alternating between co-participants B and C, whereas storyteller C does so *exclusively* by focusing entirely on co-participant A and ignoring co-participant B.

In other words, the relative success in mustering emotion contagion of “Toilet woman” may also be due to the storyteller’s expressive use of gaze radius and the intensification of inclusive gaze-focusing during the Climax, whereas the relative lack of emotion contagion in “Sad story” may be related to the storyteller’s narrow gaze radius and his exclusive gaze focus on one recipient.

#### Gesture: Gesture expressivity

As noted, gesture expressivity is measured based on the Gesture Expressivity Index (GEI), which is constructed for each gesture as a mean value from five variables. To measure whether a storyteller’s gestures gain in expressivity as they approach the Climax, the storytelling’s GEI values are fed into a linear regression; the regression’s *slope* value is taken as the metric indicative of increased or decreased gesture expressivity.

**FIGURE 11:**
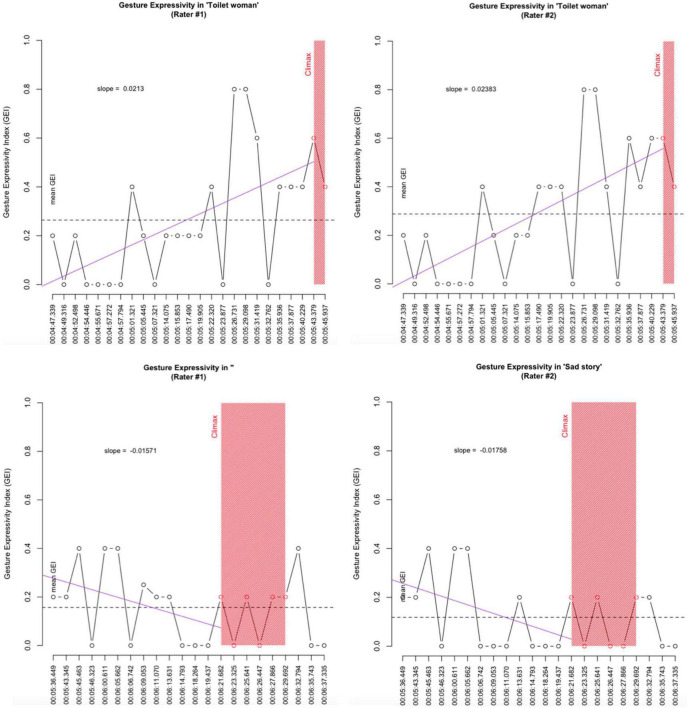
Gesture Expressivity in “Toilet woman” vs. “Sad story”. Dashed line: mean Gesture Expressivity Index. Solid pink line: regression (‘trend’) line computed from story beginning to Climax onset.

As can be seen from Table 2, rating agreements were high. As expected, the three strictly binary categories—MA, ND, and SL—saw complete rating agreement. Ratings for the two continuous variables SH and FO were remarkably close, ranging between 81% for size (SH) to 92% for force (FO). The results for gesture expressivity in the two storytellings are depicted in Figure 11.

**TABLE 2:** Agreement rates for parameters of Gesture Expressivity Index (GEI); all parameters except ND (Nucleus duration), which was computed mathematically from the durations of the stroke and, if available, hold phases, were rated by two independent raters.

Label	Variable	*N* rating diff	Agreement pct
SZ	Size	9	81.25
FO	Force	4	91.67
MA	Multi-articulator	0	100
ND	Nucleus duration	0	100
SL	Silent gesture	0	100

The comparison of the two storytellings in terms of the storyteller’s gesture expressivity yields striking differences. Not only is the storyteller’s mean GEI (overall expressivity) substantially higher for “Toilet woman” than for “Sad story” (0.29 and 0.26 vs. 0.18 and 0.14). But, more importantly, gesture expressivity develops in opposite directions. While storyteller A’s gestures gain in expressivity from story beginning to Climax, as shown by the positive slope values of 0.0213 and, respectively, 0.02383 and depicted in the trend lines in the upper two panels in Figure 11, storyteller C’s gestures lose in expressivity as he approaches the Climax, as shown by the negative slope values of −0.01571 and, respectively, −0.01758 and the declining trend lines in the lower panels in Figure 11.

For illustration, consider the respective gestures with the maximum GEI values (both storytellers have more than one maximum-GEI gesture, so we will pick the story-first each).

**FIGURE 12:**
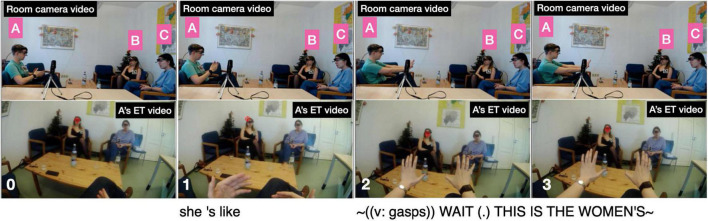
Storyteller A’s gesture during “she’s like ∼(v: gasps) WAIT (.) THIS IS THE WOMEN’S∼”.

As shown in Figure 12, storyteller A’s most expressive gesture, with a GEI value of 0.8, occurs when she describes the second cleaning lady’s denying her entrance to the women’s toilet. The gesture accompanies the constructed dialog “she’s like ∼(v: gasps) WAIT (.) THIS IS THE WOMEN’S∼” [cf. line 34 in excerpt (1)]. Prior to the gesture she has held her hands slightly above her lap (as shown in panel 0). Upon introducing the quote with “she’s like” she lifts both arms to chest height and turns the hands inward (panel 1) in preparation of the gesture stroke. In a very quick movement, on producing the gasp “(v: gasps)” she turns the palms outward, pushes the arms forward, and holds that stretched-out position as she performs the quote itself “WAIT (.) THIS IS THE WOMEN’S.” The hold is of considerable length (almost 2 s), which is why the nucleus duration is greater than story average (ND = 1). Stretching and holding out the arms for such a long time requires muscular effort (FO = 1) and covers considerable size (SZ = 1). The manual gesture is synchronized with activation of other bodily articulators (MA = 1) as the gasp is delivered with facial expression — the storyteller’s eyes and mouth are briefly wide open. The only variable that scores 0 is SL (silent gesture) as the gesture is co-speech.

**FIGURE 13:**
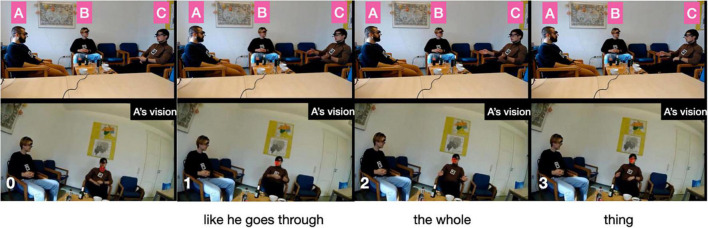
Storyteller C’s gesture during “like he goes through the whole thing”.

Storyteller C’s most expressive gesture, by contrast, has a GEI score of 0.4. It is one of the earliest gestures in the storytelling (in fact, the third) and is part of the Background section, in which he summarizes the father’s first steps toward a career as a diplomat saying “like he goes through the whole thing” [cf. line 9 in excerpt (2)]. From a rest position in his lap (pictured in panel 0 in Figure 13), he performs a movement that is sizable (SZ = 1) and requires muscular effort (FO = 1) as he starts opening both his arms laterally (panel 1), reaches an open palms oblique position, which is briefly held (panel 2) before he retracts both hands starting with the left hand first (panel 3). The nucleus, however, is not longer than average (ND = 0), the manual movement is not co-articulated with other articulating organs (MA = 0), and it is not silent but co-speech (SL = 0).

The two gestures are representative of the distinct ways the respective storytellers use, or don’t use, gesticulation expressively and in alignment with the telling’s progression toward the Climax: storyteller A’s most expressive gesture has an overall higher GEI score than storyteller C’s most expressive gesture; also, it occurs closely prior to the Climax whereas storyteller C’s gesture is deeply embedded in the Background section (as are his two other gestures with maximum GEI score; cf. Figure 11).

To conclude, as with the other multimodal parameters considered, gesture expressivity too may contribute to the differential distribution of emotional resonance across the two storytellings: in “Toilet woman” the storyteller expends great effort at intensifying the expressiveness of her gestures as she progresses toward the Climax while in “Sad story” the storyteller’s gestures progressively lose in expressivity.

Having thus concluded the discussion of the multimodal analyses it is time for a ‘but.’ But what about recipient C in “Toilet woman” — why is she not affected by the storyteller’s multimodal mobilization of arousal and contagion? And what about recipient B in “Sad story” — why is his EDA response not only not in line with recipient’s A response but on a downward trajectory? And why, finally, do we have a consistent production of engaged response tokens around the Climax suggesting emotion contagion when in fact that contagion, if any, is inconsistently distributed across the recipients? To address these questions we need to look beyond the multimodal performance by the storyteller and instead consider factors beyond his/her control.

### Beyond the storyteller’s control

#### Participation framework

A first factor to consider is participation framework. In “Sad story,” we saw an unequal distribution of storyteller C’s gaze toward recipient A. The reason is simple: recipient B is storyteller C’s brother, making C and B ‘family’ and, thus, one ‘party,’ a unit of social organization “which can be claimed to have a persistence and reality quite apart from the interaction” (Schegloff, 1995: 33). By not addressing his gaze to him during the story about another member of their family, storyteller C demonstrably treats B as a coincumbent of the same party. Given his status as a coincumbent of the same party it makes intuitive sense to not expect to see emotional arousal in B.

#### Relevance

Another critical aspect is relevance. A broad consensus exists in the affective sciences that “in order for a particular object or event to elicit an emotion, that object or event needs to be […] *relevant* to the person in whom that emotion is elicited” (Wharton et al., 2021: 260; original emphasis). Indeed, emotions can be seen as “relevance detectors” (Scherer, 2005: 701). Relevance is unequally distributed across the two storytellings.

Being a first-person story, in which the storyteller is “the central actor or affected person” (Norrick, 2000: 149), “Toilet woman” is centrally about the storyteller who is physically co-present with the interlocutors; being third-person, “Sad story” is centrally about a person absent from the telling situation. Moreover, “Toilet woman” is about the distress the storyteller is experiencing now, in her daily life, whereas “Sad story” is about the difficulties a story character was facing a long time ago (given the age of the storyteller in his mid-20s, up to two decades!). These differences greatly increase the relevance of “Toilet woman” but diminish the relevance of “Sad story.” As a result, in their quest for emotion contagion, the two storytellers start from two distinct points: the relevance of storyteller A’s story is immediate, whereas the relevance of storyteller C’s story is remote to the point of even being questionable (after all, despite the father’s early struggles, the storyteller and his brother have developed into two young men without any apparent deficits). As a story about the distress a non-co-present person experienced at a remote time, “Sad story” requires, and invites, much less co-construction from the recipients, serving much less as an experiment for collaboratively making sense of problematic experiences (cf. Ochs and Capps, 2001: 2). As a story about the struggles a co-present person experiences in the here-and-now, “Toilet woman” essentially functions to collaboratively “air, probe, and otherwise attempt to reconstruct and make sense of actual and possible life experiences” (Ochs and Capps, 2001: 7). As such, it seeks and necessitates co-construction by the recipients, including their emotional involvement.

## Discussion and concluding remarks

This pilot study aimed to provide a first sketch of the outlines of the vast field of factors influencing emotional resonance in storytellings of sadness/distress using an extended methodology.

While nothing of what was found is definitive, a lot is suggestive. Crucially, the analyses suggest that whether, and to what extent, emotional resonance is achieved depends on a large range of factors, many under the storyteller’s control *via* the multimodal resources they use, some lying beyond their control. Success at achieving emotional contagion depends on how the controllable multimodal factors are manipulated and whether the non-controllable ones work, or do not work, to the storyteller’s advantage. Generally, multimodal *effort* helps: more use of quotation, gesture expressivity, voice modulation and gaze focusing increases the odds of sparking an emotional response in the story recipients. By contrast, it would be a waste of multimodal effort if you attempted to tell your children for the umpteenth time of an exciting event back in your heydays. The analyses also suggest the possibility that in storytellings where emotion contagion does happen, the storyteller’s multimodal effort is *synchronized* with the storytelling’s progression toward the Climax: multimodal resources are deployed climactically such that they peak when the telling peaks reaching its key event, thus illuminating it brightly so that the event will be recognized as the key event at which displays of emotional contagion are relevant. In other words, the analyses suggest the Multimodal Crescendo Hypothesis: storytellers climactically increase their multimodal efforts toward the Climax. Future work will test this hypothesis.

The analyses also suggest that future work will have to reckon with large amounts of variability in (internal) EDA and how it relates to (external) displays of emotion. EDA variability is to be expected, given that EDA is known for inter-individual variability. But that variability may compounded by mismatches with what is demonstrably communicated. In the present research, a mismatch was observed between EDA response and emotion display: all four recipients produced evaluative assessment tokens issued around the respective Climaxes but only two of them seemed to resonate emotionally (recipient B in “Toilet woman” and, to a much lesser degree, recipient A in “Sad story”). The absence of emotional excitation in two of the four recipients is a reminder that the relation between emotion and emotion display is often not one-to-one: “there can what appears to be expression without emotion” (Ekman, 1999: 48) and people can make use of “intentional, strategic signaling of affective information (…) which has no automatic or necessary relation to ‘real’ inner affective states” (Caffi and Janney, 1994: 328). The mismatch calls into question CA’s understanding of empathy. Kupetz (2014: 4) defines empathy as “displays of understanding of the other person’s emotional situation.” This definition stresses the (outwardly manifest) *display* of emotional understanding, what is otherwise referred to as ‘emotion recognition’ by Ekman (2014) or ‘cognitive empathy’ by Lawrence et al. (2004) Considering the EDA evidence presented in this paper, it appears that conceptualizing empathy in Kupetz’s way cannot account for staged and strategic emotion displays and cannot tell emotional recognition apart from emotional resonance. Considering CA’s increased interest in emotion, CA-inspired research into emotion in interaction may therefore benefit from embracing psychophysiological measures.

If, then, displays of emotional resonance cannot be trusted to index shared emotion, the question arises whether *participants* trust displays of emotional resonance. We know they will pursue them when they are relevantly missing (s. Introduction). What if the resonance display obtained (with or without pursuit) is not carried by experienced emotion—will they notice and/or will they care? Investigating affiliation and emotional arousal in storytellings, Peräkylä et al. (2015) noticed a pattern of recipient affiliation leading to a decrease in the storyteller’s arousal and an increase in the recipient’s arousal (Peräkylä et al., 2015: 318), a pattern they refer to as ‘sharing the emotional load.’ If storytelling is all about sharing the emotional burden, then displays of empathy that hide a lack of experienced empathy might not be enough—storytellers might indeed notice and care! But more work into the sharing-the-burden pattern is needed before we can answer these questions definitively.

The study was designed as a pilot study to stake out interim directions for future work. As such it carries with it the burden of multiple limitations. The first, most obvious limitation is the limited size of the data scrutinized. Needless to say that there is more to the story of emotion contagion than analysis of two stories can tell. A pressing desideratum for future research is therefore the compilation of a collection of storytellings that is substantially larger in terms of number of stories included.

The second limitation is that no inferential statistical tests were used. Given that only two storytellings were examined, that decision was warranted. But we have no way to reliably estimate how representative the two storytellings are. Future work based on data of scale will therefore inevitably have to use rigorous statistical modeling.

The third limitation is that despite the already-large number of factors that were considered here, that number is likely not large enough. For example, storytellers’ multimodal behavior may be governed by (invariable) personality traits. Among the Big Five traits (extroversion, emotional stability, agreeableness, conscientiousness, openness to experience; cf. Goldberg, 1990) extroversion (v. introversion) has been shown to correlate with more, faster and louder talk (e.g., Argyle, 1988), faster and more sizable gestures (e.g., Mehl et al., 2007) as well as more direct eye contact (Riggio and Friedman, 1986). The question arising, then, is whether and to what extent extroverts stand a better chance of mustering emotional resonance than introverts, who tend to talk less, use longer pauses and more hesitations, produce fewer and less expansive gestures and make less eye contact.

Another potential source of contagion-related influence not considered here is how susceptible participants are to emotional contagion in the first place. Susceptibility to emotion contagion is defined as “the tendency to automatically mimic and synchronize with the expressions of others and, through afferent feedback from the facial and/or skeletal muscular activity, to experience or ‘catch’ the others’ emotions” (Doherty, 1997: 149). A wide range of factors may contribute to individual differences in susceptibility to emotional contagion, among them genetics, early experience, and personality characteristics (Doherty, 1997: 133). Obviously, most of these factors will be virtually impossible to control for in any research setting. The only factor that may contribute to individual differences in susceptibility and that can be controled for is gender. But researchers are not in agreement whether gender is correlated with susceptibility. In a large meta-study, females did not exhibit more empathy than males when physiological measures were used to index empathy (Eisenberg and Lennon, 1983:124). In Doherty (1997), by contrast, women were highly significantly more susceptible to emotion contagion than men (Doherty, 1997: 140).

The list of factors impacting contagion will most likely increase even further once more data is scrutinized. Taking large numbers of factors into account is doubtlessly a daunting challenge. But given that emotion is the heartblood running through the veins of storytelling, and that sharing emotion creates a strong sense of connectedness that is essential not only to human cooperation but also health (Sterling, 2012), taking up the challenge is highly desirable.

The present research has taken the first step toward answering that challenge. Clearly, more steps will have to follow to gain a deeper and more comprehensive understanding of how participants to storytelling in conversational interaction share, and fail to share, their emotions.

## Data availability statement

The raw data supporting the conclusion of this article will be made available by the authors, without undue reservation.

## Ethics statement

Ethical review and approval was not required for the study on human participants in accordance with the local legislation and institutional requirements. The patients/participants provided their written informed consent to participate in this study. Written informed consent was obtained from the individual(s) for the publication of any potentially identifiable images or data included in this article.

## Author contributions

The author confirms being the sole contributor of this work and has approved it for publication.

## Footnotes

1 Cf. Bar’s (2009) ‘recognition-by-analogy’ process.

2 For an authoritative discussion of the various ways in which ‘altruism’ has been conceptualized and whether there is ‘true’ altruism that does not ultimately serve a self-interest, see Batson (1987).

3
https://gepris.dfg.de/gepris/projekt/414153266

4 As remarked by an reviewer, “a single story can evoke or involve many different emotions.” This is certainly true. The stories under investigation here, however, can be said to *centrally* revolve around sadness/distress because it is this emotional spectrum that is both signposted as relevant by the storyteller, verbally or non-verbally, and the one responded to by the recipients at or around the respective Climax.

5 EDA can also be indicative of attentional and cognitive processes (Dawson et al., 2000).

6 The phasic EDA response type is often also referred to as galvanic skin response (Lykken and Venables, 1971).

7 The interrater agreements in Dael et al. (2013) were expecially low for fluency and height.

8 The fine-grained detail with which facial expression is studied, for example, in Eisenberg et al. (1989) is however unattainable in this research, where the somewhat bulky eye tracking glasses hide much of the facial cues around eyes, eyebrows, and forehead. This is deplorable because most of the facial cues accompanying the emotion of sympathy are centered precisely around these parts of the face (Eisenberg et al., 1989: 58).

9 Psychopathological states have a fundamental effect on electrodermal responsivity, with schizophrenia and depression often going hand-in-hand with stimulus hyporesponsivity (cf. Dawson et al. (2000) and references therein).

10 Malfunctioning of the subject’s wrist watch can be excluded as the subject did show responsivity in later stages of the conversation.

11 If we say that silent quotes depict a *meaning* rather than a gesture, we leave open which semiotic modality that meaning was conveyed through, be it verbal, gestural, facial, or any other modality, or whether it was not conveyed at all (but, for example, part of internal dialog).
